# Effectiveness of Community Nutrition-Specific Interventions on Improving Malnutrition of Children under 5 Years of Age in the Eastern Mediterranean Region: A Systematic Review and Meta-Analysis

**DOI:** 10.3390/ijerph18157844

**Published:** 2021-07-24

**Authors:** Delaram Ghodsi, Nasrin Omidvar, Bahareh Nikooyeh, Roshanak Roustaee, Elham Shakibazadeh, Ayoub Al-Jawaldeh

**Affiliations:** 1 Department of Nutrition Research, National Nutrition and Food Technology Research Institute and Faculty of Nutrition Sciences and Food Technology, Shahid Beheshti University of Medical Sciences, Tehran 1981619573, Iran; delaramghodsi@yahoo.com; 2 Department of Community Nutrition, National Nutrition and Food Technology Research Institute and Faculty of Nutrition Sciences and Food Technology, Shahid Beheshti University of Medical Sciences, Tehran 1981619573, Iran; r_roustaee@yahoo.com; 3 Laboratory of Nutrition Research, Department of Nutrition Research, National Nutrition and Food Technology Research Institute and Faculty of Nutrition Sciences and Food Technology, Shahid Beheshti University of Medical Sciences, Tehran 1981619573, Iran; nikooyeh11024@yahoo.com; 4 Department of Health Education and Promotion, School of Public Health, Tehran University of Medical Sciences, Tehran 1417913151, Iran; shakibazadeh@tums.ac.ir; 5 Nutrition Unit, World Health Organization Regional Office for the Eastern Mediterranean, World Health Organization, Cairo 11371, Egypt; aljawaldeha@who.int

**Keywords:** community-based intervention, nutrition-specific intervention, malnutrition, EMR countries, children, effectiveness

## Abstract

Childhood malnutrition remains an important public health and development problem in low- and middle-income countries. This study aimed to systematically review the community-based nutrition-specific interventions and their effectiveness and/or cost-effectiveness on the nutritional status of children under 5 years of age in the Eastern Mediterranean Region (EMR). A systematic literature search of the English electronic databases, including PubMed, Scopus, ISI Web of Knowledge, Ovid, EMBASE, as well as Persian databases (SID and Magiran) was performed up to May 2019. Studies regarding the effectiveness/cost-effectiveness of the community-based nutrition-specific programs and interventions targeted at under-five-year children in EMR countries were selected. The primary outcomes were mean of Weight-for-age z-score (WAZ), Height-for-Age z-score (HAZ), and Weight-for-Height z-score (WHZ) of children or prevalence of wasting, stunting, and/or underweight among the children. Meta-analysis was also performed on the selected articles and intervention effects (mean differences) were calculated for each outcome for each study and pooled using a weighted random effects model. Risk of bias (ROB) of each included study was assessed based on the Cochrane Handbook for Systematic Reviews. The study protocol was registered in PROSPERO (CRD42020172643). Of 1036 identified studies, eight met the inclusion criteria. Amongst these, seven were from Pakistan and one from Iran. Only one study conducted in Pakistan reported the cost-effectiveness of nutrition-specific interventions in the region. Nutrition education/consultation and cash-based interventions were the most common nutrition-specific strategies used for management of child malnutrition in the EMR countries. Out of these eight studies, four were included in the meta-analysis. When different interventions were pooled, they had resulted in a significant improvement in WHZ of children (MD: 0.26; 95% CI: 0.07 to 0.46, three studies, I^2^ 82.40%). Considering the high prevalence of child malnutrition in a number of countries in the region, capacity building and investigation regarding the implementation of new approaches to improve nutritional status of children and their effect(s) and cost-effectiveness assessment are highly recommended.

## 1. Introduction

Childhood malnutrition remains an important public health and development problem in low- and middle-income countries (LMICs) and regional, sub-regional, and country disparities in undernutrition remain [1]. Recent studies have shown that childhood undernutrition affects children’s health and growth and their cognitive abilities and productivity in adulthood, with measurable economic impacts [2]. This is why, ending all forms of hunger and malnutrition has been targeted as one of the Sustainable Development Goals (SDGs), explicitly referencing chronic child malnutrition [3]. There is an explosion of evidence on long-term health and economic benefits of improved nutrition in early childhood and as a result, most interventions have focused on the early lifecycle stages [4,5,6]. Even though the fact that addressing childhood undernutrition is one of the approaches that can result in increased individual well-being, as well as overall economic growth, cost-effectiveness/cost–benefit of different strategies needs to be measured in different context.

Child malnutrition causation depends on a range of immediate, underlying, and basic determinants and their interactions [7]. Nutrition-specific interventions that address the immediate determinants of child malnutrition [8] are being used for improvement of child malnutrition in different contexts. Studies have shown that the scaled-up coverage of nutrition-specific interventions is crucial for undernutrition reduction [9,10]. 

The Eastern Mediterranean Region (EMR) of the World Health Organization (WHO) comprises 22 countries extending from Pakistan in Southern Asia to Morocco in North Africa. These countries are ecologically, economically, and socially very different and at various stages of development. There are also differences in health and nutrition situation and achievements in combating malnutrition and promoting health and nutrition of the people among these countries [11]. The EMR has witnessed significant social, economic, and political changes that have impacted lifestyle and health profile of the population and child’s nutritional status over the past few decades [12,13]. In 2004, a review on maternal and child malnutrition in the region showed that the proportion of underweight, wasted, and stunted children has had a downward trend till the last decade [11]; and the prevalence of wasting and stunting has been increasing gradually since 2006 [14,15,16]. Additionally, a review of nutritional status in Arab countries showed the paradox of two contrasting nutrition-related diseases existed: those associated with inadequate intake of nutrients and unhealthy dietary habits, i.e., growth retardation among young children and micronutrient deficiencies; and those associated with changes in lifestyle, including obesity and diet-related non-communicable diseases [17]. Similar results have been reported in other countries of the region, where nutrition transition is taking place, including Iran, Egypt, Lebanon, and the United Arab Emirates [13,18,19,20]. Another review from six selected countries in the region showed different ranges of wasting (1.1 to 11.8%), stunting (7.3 to 9.3%), and underweight (1.6 to 5.3%) across these countries [13]. In the meantime, there are many children in the region who are suffering from poor nutrition because of war and conflicts, as well as poverty, specifically in Iraq, Syria, Yemen, Palestine, and Afghanistan [21,22,23]. A number of efforts have been made to combat child malnutrition in the region. Despite nutritional and non-nutritional interventions and programs implemented for alleviating poverty and improving the nutritional status of children in the region, not much published information is available [11]. A recent scoping review also showed that one of the major gaps in nutrition-related research in Arabic countries was the limited available evidence among children [24].

Allen and Gillespie in 2001 through a review on the nutrition interventions in several countries, including some of the EMR countries, showed that large-scale nutrition interventions could potentially affect child malnutrition [25]. The effectiveness of each of these interventions on child malnutrition depends on the community needs and the context and initial situation. To the best of our knowledge, there has been no systemic review and meta-analysis assessing the effects/cost-effectiveness of nutrition-specific interventions targeted children under 5 in EMR. Therefore, this systematic review was designed to identify available nutrition-specific interventions targeted at under-5 child malnutrition in the EMR countries and their effectiveness and/or cost-effectiveness as a basis for planning future interventions. The result can provide a general picture of efforts being made on this regard, as well as potential gaps; and can provide take home lessons for future planning of programs to combat malnutrition in the region’s countries.

## 2. Materials and Methods

### 2.1. Search Strategy

The population, intervention, comparator, and outcome (PICO) questions addressed in this study were framed as follows: what is the effectiveness/cost-effectiveness of community-based nutrition-specific interventions on nutritional status of children under 5 as compared to those who did not receive such intervention in the EMR countries. In the first step, key terms regarding nutrition-specific interventions [8] were identified. A comprehensive search strategy was designed and applied to English electronic databases, including PubMed, Scopus, ISI Web of Knowledge, Ovid, EMBASE, as well as Persian-language databases (SID and Magiran). We used keywords and search terms relevant to EMR (Afghanistan OR Bahrain OR Djibouti OR Egypt OR Iran OR Iraq OR Jordan OR Kuwait OR Lebanon OR Libya OR Morocco OR Pakistan OR Oman OR Palestine OR Qatar OR Saudi OR Somalia OR Sudan OR Syrian OR Tunisia OR Emirates OR Yemen OR EMRO OR MENA), nutrition-specific interventions including breast feeding promotion, complementary feeding, and increasing food access (AND (nutr* OR food* OR diet* OR feed* OR ration* OR cash* OR ration OR in-kind* OR distribut* OR basket* OR cash* OR voucher* OR aid), malnutrition and growth (grow* OR weight* OR height* OR leng* OR waz OR haz OR whz OR malnut*), and children (infant* OR preschool* OR toddler* OR child*). For the Persian database, similar words in Farsi have been used. References of retrieved records, relevant reviews, and systematic reviews were scanned for potentially eligible studies. 

The study was designed as a systematic review according to the Preferred Reporting Items for Systematic Reviews and Meta-Analyses (PRISMA) statement (see checklist in Supplementary File S1). The protocol of the study was completed before commencement of the study and submitted within International prospective register of systematic reviews (PROSPERO). Registration number is CRD42020172643.

### 2.2. Study Eligibility Criteria

In this review, the community-based nutrition-specific interventions in English and/or Persian language without any time limitation that investigated promotion of optimum breastfeeding, complementary feeding and responsive feeding practices and stimulation, mother’s education on complementary feeding, and interventions that increase the accessibility of foods/beverages for children, take home food packages or food vouchers or any kinds of cash transfers targeted child nourishment in combination with or without child supplementation (micronutrient supplementation, food fortification) targeted malnutrition and growth of children under five in EMR countries were included. This review considered randomized controlled trials (RCTs), cluster randomized, quasi-randomized, and non-randomized controlled trials, controlled before and after studies (CBAs), and interrupted time series (ITS) aiming at children less than 5 years of age in the EMR countries and were published up to 31 May 2019 in English and/or Farsi languages. The primary outcome measures included child nutritional status, determined by anthropometric indicators, including: mean of Weight-For-Height/Weight-For-Length Z-score (WHZ/WLZ), Weight-For-Age Z-score (WAZ), and Height-For-Age/Length-For-Age Z-score (HAZ/LAZ) or prevalence of underweight, stunting, and wasting.

Interventions providing micronutrients alone and those distributed commercial products for the purpose of treatment and hospital-based interventions were outside the scope of this review.

### 2.3. Article Screening and Data Extraction

Titles and abstracts of the identified papers were screened by two authors, independently. The studies selected at the first stage, underwent a full-text screen against the second stage inclusion and exclusion criteria. For each article, two reviewers independently reviewed the full text. Disagreements regarding eligibility were resolved by discussion and consultation with a third reviewer. Quality assessment was done after completion of screening by three authors.

For each of the included studies, the following information was tabulated independently by two reviewers using a standardized data extraction form: author and year of the study, setting, study design, subjects and sample size, kind of intervention and its duration, effect/outcome of the study, and cost/cost-effectiveness of the intervention. Resources that were not related to nutrition-specific intervention targeted at under-5-year-old children and those did not report the inclusion criteria for outcome were excluded from the study. To avoid mistakes due to data manipulation, we first collected data as they were reported and only subsequently, performed transformations. No major data transformation was needed, except for calculation of the differences of means in some studies. In the two cases that data were missing or unclear, we attempted to contact the authors to clarify relevant and missing data.

### 2.4. Risk of Bias Assessment

Methodological quality and risk of bias (RoB) were assessed by three reviewers independently based on the Cochrane guidelines for RCTs, using Cochrane RoB tool [26] by two authors for each of the included full-texts. Five types of RoB for randomized trials, including selection, performance, attrition, detection, and reporting biases were examined. Intervention studies involving animals or humans, and other studies that require ethical approval, must list the authority that provided approval and the corresponding ethical approval code.

### 2.5. Data Synthesis and Meta-Analysis

Meta-analysis was conducted to obtain the mean difference of the various outcomes of interest, including WAZ, HAZ, and WHZ to measure the magnitude of the treatment effect of interventions. For the meta-analysis, trials were grouped by the type of intervention. For multi-armed studies, pairs of arms relevant to the review were compared. Data for the control group were used for each intervention group comparison. The weight assigned to the control group was reduced by dividing the control group number (N) by the number of intervention groups. In the present study, subgroup analysis could not be applied due to the limited number of included studies in each intervention group. Therefore, it was not possible to explore probable differences between studies by age of the children and type of interventions. Depending on data availability, an exploratory subgroup analysis for duration of intervention (duration ≤6 months and >6 months) was conducted.

### 2.6. Meta-Regression

There were not enough RCTs to conduct a meta-regression.

### 2.7. Statistical Analysis

Meta-analysis was performed using STATA software, version 16.0 (StataCorp, College Station, TX, USA). We did not report any dichotomous data. For continuous outcomes, a mean difference (MD) and 95% CIs was calculated for each study (i.e., intervention group minus control group differences). In addition, heterogeneity was assessed using Q test and I^2^ test. The fixed effect model was used when there was no statistically significant heterogeneity (*p* > 0.1 and I^2^ < 50%), whereas a random-effects model was employed on the contrary (*p* < 0.1 or I^2^ > 50%).

## 3. Results

**Figure 1:**
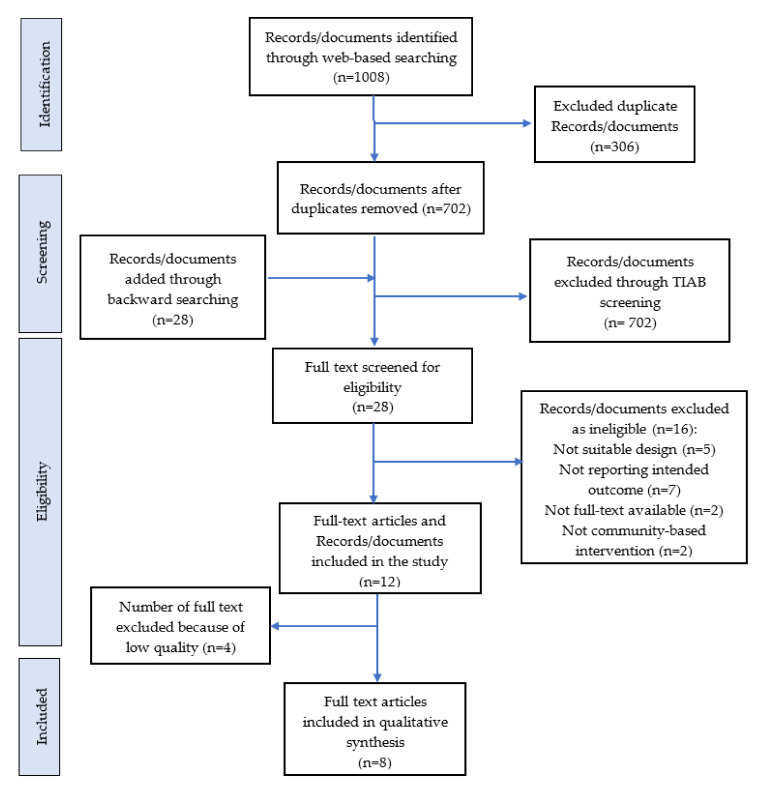
PRISMA diagram of information flow through phases of the systematic review.

The results of searching and resource selection procedures are presented in Figure 1, based on the Preferred Reporting Items for Systematic Reviews and Meta-analyses (PRISMA) checklist. As presented in Figure 1, out of 1036 studies identified through electronic search, hand searching, and data received from the region in the first stage of the systematic review, 1040 were excluded due to being irrelevant based on their titles and/or abstracts or duplication between databases. Of 28 remaining articles, 16 were excluded following the full-text assessment of eligibility due to unclear or ineligible intervention, not reporting the intended outcome, and/or unfavorable study design (Table 1). 

Low quality was the reason for further exclusion of four articles (Table 2). Finally, eight articles remained that met the inclusion criteria for the systematic review (Table 3). Amongst them, only four [27,28,29,30] could be included in meta-analysis. 

Seven of the included studies were from Pakistan and one from Iran. Only one study that was conducted in Pakistan had evaluated cost-effectiveness of nutrition-specific interventions for improvement of nutritional status of children. The selected studies were also conducted in various contexts, including urban and rural areas. The results are presented in two sections: (1) a descriptive systematic literature review and (2) a meta-analysis which conducted to estimate a pooled effect size.

### 3.1. Descriptive Systematic Literature Review

Table 3, presents a summary of the characteristics of accepted studies, including the nature of the interventions, the design and some outcomes. The interventions were categorized based on the main nutrition-specific strategies into two categories: nutrition education/counselling and provision of food through different modalities.

#### 3.1.1. Nutrition Education/Nutrition Counselling

Five studies had used nutrition education approaches regarding child feeding as the main intervention and child growth rate as one of the primary outcomes (Table 3). They were all designed as effectiveness studies, conducted in Pakistan, and delivered within health centers or routine health care facilities through lady/community health workers. In four studies, educational massages were directly provided to the mothers and integrated into existing routine home visits [27,28,29,51]. In one of the interventions conducted in Karachi, trained community health workers or volunteers were trained on nutrition consultation skills with mothers, child growth monitoring, and complementary feeding and the impact of this intervention was observed on the care and feeding practices of mothers referred to them [50]. In the three others that were all related to one intervention program [27,28,51], nutrition education was complemented with the provision of micronutrient powder among targeted children. In all the included studies, the control group received routine nutritional and health services. Except for two studies that followed children after two years of age [27,51], all interventions targeted children through the first two years of life [27,28,29,50,51]. The main nutritional messages in all the studies in this category were focused on proper breastfeeding (BF) and its importance, initiation of complementary feeding [CF) at six months of age, promoting protein-based, iron-rich foods, and dietary diversity. 

In the study by Saleem et al., the children were from relatively food-secure populations, and the results showed that educational interventions about appropriate CF to mothers directly affected linear growth and reduced stunting, wasting, and underweight of their children [29]. Zaman et al. [50] reported a significant effect of nutrition education on WAZ, while no significant effect was seen on the mean of length-for-age. Feeding practices were the other outcome considered in this study. The proportion of mothers reporting to offer eggs and meat to their children were significantly higher in the intervention group than in the controls (*p* = 0.03 and *p* = 0.01). 

In three studies in which children received nutrition education and multiple micronutrient powders (enhanced nutrition) [27,28,51], HAZ was significantly improved at six months and 18 months compared to children not exposed to enhanced nutrition. Mean WAZ did not differ significantly between the groups at 6, 12, 18, or 24 months [28]. At the follow-up of the children at age 4, no significant differences were observed in the proportion of children who were underweight, stunt, and wasted across groups [27]. 

#### 3.1.2. Food Distribution through Different Modalities

Two studies in the region reported the effectiveness of the provision of food through cash, voucher, or in-kind food(s) on improving the nutritional status of children [30,46] and one was related to the cost-effectiveness of this category of interventions [47]. In both effectiveness studies, the primary outcomes considered were improvement or changes in anthropometric indices [30,46]. In Iran, the effects were evaluated for six months, but in Pakistan, an evaluation study conducted for one year. All of these studies targeted children under 5 years of age from poor households [30,46,47].

In Iran [30], three modalities of supplementary food distribution have been implemented, including in-kind, food vouchers, and electronic cards (E-cards), and the value of the food basket was similar in different distribution modalities, while in Pakistan [46], different values of Cash-Based Interventions (CBIs) were delivered. Ghodsi et al. showed changes in HAZ were greater in those who received supplementary foods by food voucher, while there were no significant differences in WAZ and WHZ by different distribution modalities [30]. In Pakistan [46], significant differences in the primary outcome were seen only at six months and double cash (DC) transfer was the effective intervention in improving WHZ. The only study that reported the cost, cost-efficacy, and cost-effectiveness of the CBI in the region was conducted in Pakistan. It was related to the economic evaluation of different cash transfer interventions [47]. The DC was the most cost-efficient intervention, followed by the SC (standard cash), and finally the FFV (fresh food voucher). However, when the cost of participation to beneficiaries was deducted from the amount transferred, the FFV was more cost-efficient than the SC, indicating that the inclusion of costs to beneficiaries is essential for an accurate estimation of overall cost-efficiency. These three interventions were highly cost-effective by international thresholds. 

### 3.2. Meta-Analysis

For the meta-analysis, out of eight included studies in the systematic review, three [29,46,47] were excluded because of not reporting the mean or prevalence of outcome at follow-up. Despite the emails sent to the corresponding authors and requested the required data, no response was received. One of these studies [47] was cost-effectiveness analysis of the other one [46], which was also excluded. These studies varied in size, duration, and intervention type.

The effect sizes of all the five remaining studies were combined to estimate an overall summary effect size (and 95% confidence interval) for WAZ, HAZ, and WHZ by using a random effects meta-analysis model. 

#### 3.2.1. Impact of Different Nutrition-Specific Interventions on HAZ

**Figure 2:**
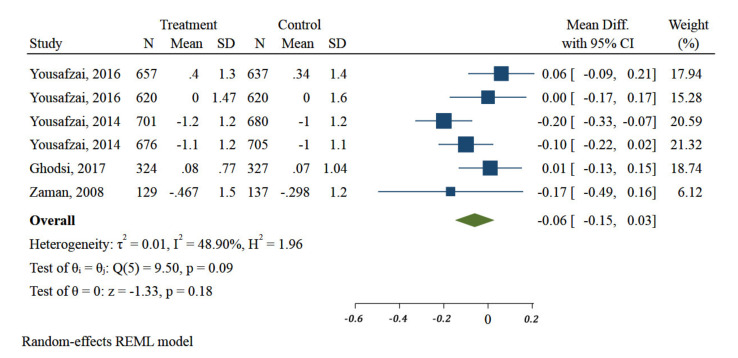
Impact of different community-based nutrition interventions on Height-for-Age Z-score of children under 5 in Eastern Mediterranean Region.

When the results of various interventions were pooled together, there was no evidence of improvement of children HAZ after interventions (MD: −0.06; 95% CI: −0.15 to 0.03, I^2^ 50%) (Figure 2).

**Figure 3:**
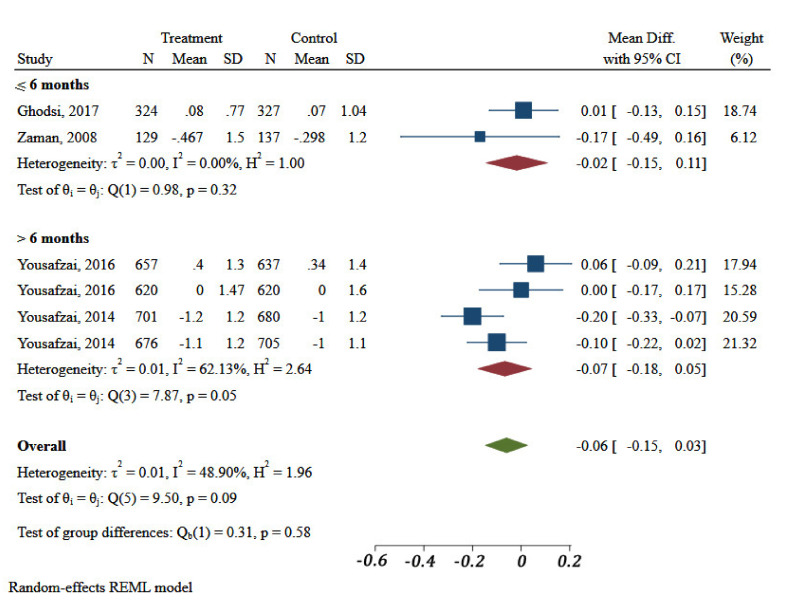
Impact of different community-based nutrition intervention duration on Height-for-Age Z-score of children under 5 in Eastern Mediterranean Region.

Duration of intervention in two of the four included studies was less than or equal to 6 months. Meta-analysis showed no differences in HAZ of children in the included interventions by their duration (*p* = 0.58) (Figure 3).

#### 3.2.2. Impact of Different Nutrition-Specific Interventions on WAZ

**Figure 4:**
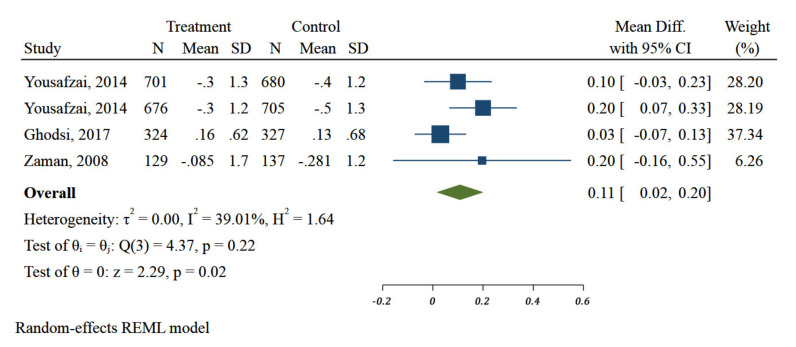
Impact of different community-based nutrition interventions on Weight-for-Age Z-score of children under 5 in Eastern Mediterranean Region.

**Figure 5:**
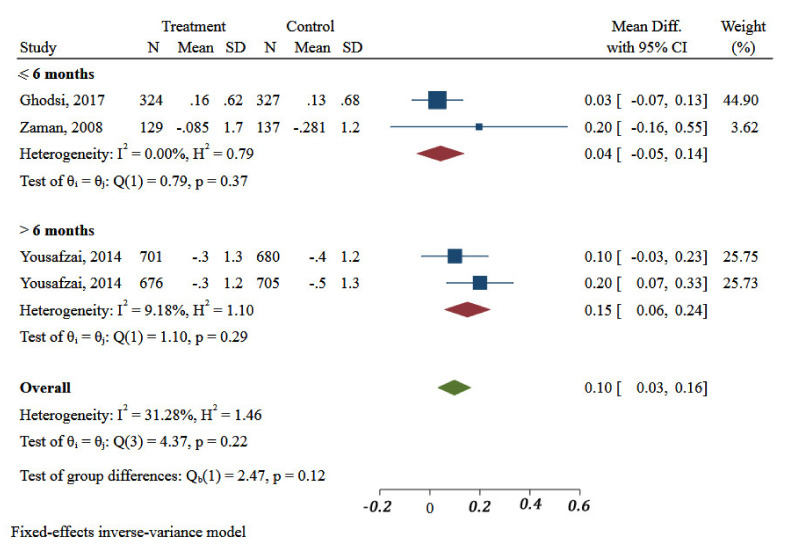
Impact of different community-based nutrition intervention duration on Weight-for-Age Z-score of children under 5 in Eastern Mediterranean Region.

Taking all of the studies together, nutrition intervention strategies (education and extra food distribution), had a significant effect on WAZ (MD: 0.11, 95%CI: 0.02 to 0.2; three studies, I^2^: 39.01%) (Figure 4). Duration of intervention in one (two arms) of three included studies was more than or equal to 6 months. Meta-analysis showed significant differences in duration of interventions on improvement of WAZ of children in the EMR (MD: 0.04; 95%CI: −0.05 to 0.14 vs. 0.15 95%CI: 0.06 to 0.24, *p* = 0.12) (Figure 5).

#### 3.2.3. Impact of Different Nutrition-Specific Interventions on WHZ

**Figure 6:**
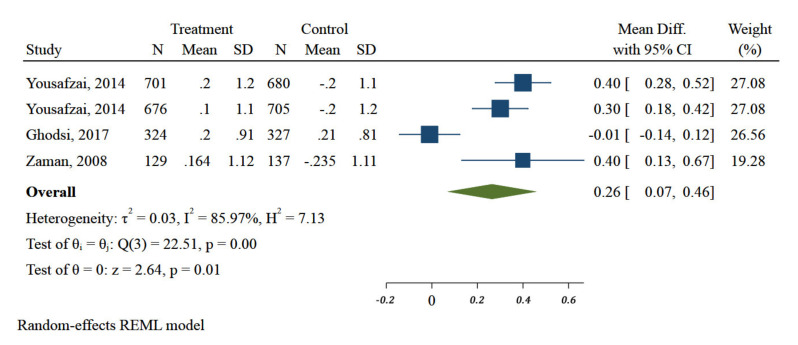
Impact of different community-based nutrition interventions on Weight-for-Height Z-score of children under 5 in Eastern Mediterranean Region.

There was a significant difference in improvement of WHZ of children when different interventions were polled together (MD: 0.26; 95% CI: 0.07 to 0.46, three studies, I^2^ 82.40%) (Figure 6). 

**Figure 7:**
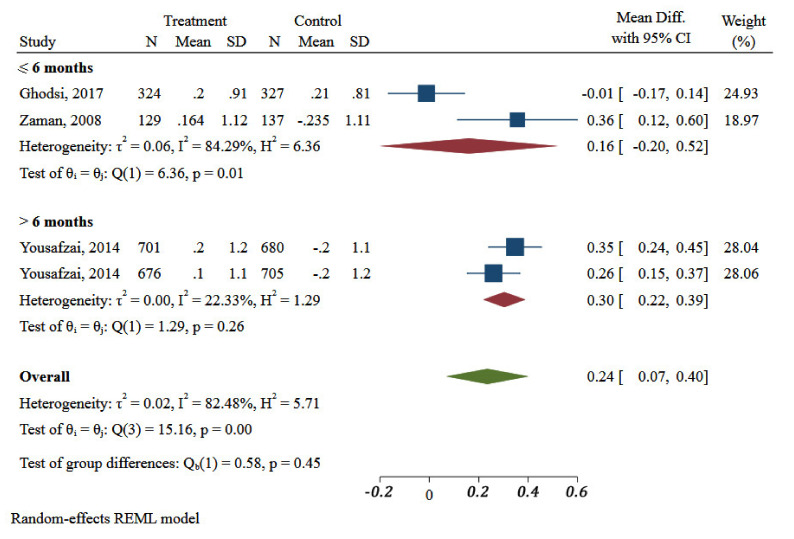
Impact of different community-based nutrition intervention duration on Weight-for-Height Z-score of children under 5 in Eastern Mediterranean Region.

Subgroup analysis showed significant differences in duration of interventions and improvement of WHZ of children. Intervention lasted more than 6 months had significant effects on improvement of the WHZ status (MD: 0.35; 95% CI: 0.25 to 0.45, one study, I^2^ 22.33%) (Figure 7).

## 4. Discussion

The purpose of this systematic review and meta-analysis was to identify and summarize available information on the effect of community-based nutrition-specific interventions on improving the nutritional status of children under 5 in the EMR countries. Nutrition education and counselling on child complementary feeding and providing food through cash transfer, vouchers or as in-kind were the main nutrition-specific strategies used for the improvement of nutritional status of children in the region. When all studies were pooled together, the interventions had a significant effect only on WHZ status of children. 

All the identified interventions had been integrated into routine health care, including breastfeeding promotion, immunization, water, sanitation and hygiene (WASH), and growth monitoring and promotion. Hossain et al. also showed that interventions routinely delivered through health system and social safety nets were the most commonly implemented interventions in most LMICs and were effective in reducing stunting [54]. 

Nutrition education and counselling were among the main strategies used in almost all the included studies and targeted either health workers [29] or mothers [50]. Both of these approaches had significant effects on the improvement of anthropometric indices of the children. This finding is consistent with previous reviews that showed that community-based nutrition education interventions effectively improve the nutrition status of children under five in developing countries and their linear growth and weight gain [55,56,57]. 

Besides, it is shown that in-service nutrition training [58] and a carefully selected, small number of specific key messages about feeding practices feeding [59] are more effective in managing nutrition-related conditions, especially child undernutrition. One of the important take-home lessons from the present review is that nutrition education can have significant effects on the improvement of weight and height status of children even alone if implemented in a relatively food secure context. There is evidence on the significant impacts of nutrition education on the child growth indicators in the poor; however, sustainability of the impacts on children’s nutritional status even with the micronutrient powder is under question. In line with these results, a systematic review of studies in LMICs, also showed that nutrition education interventions are highly context-specific. In a population with higher food insecurity, complementing it with provision of a food supplement is recommended (59]. After all, we should note that nutrition education and supplementary food distribution, by themselves, cannot eliminate the underlying causes of child malnutrition, i.e., poverty and poor sanitation, and they should be addressed through appropriate policies and interventions [59]. 

Increasing access to foods was the other nutrition-specific strategy being used in the included studies. Three studies, one from Iran [30] and two from Pakistan [46,47], evaluated the effect/cost-effectiveness of food distribution programs for the nourishment of children under 5. Some of the reasons identified for low effectiveness of this intervention in Iran were low nutrient density of the food items, including the food basket and inconsistency between the food basket items in the program and foods selected by the beneficiaries in the defined stores. Food sharing between the family members because of poverty and low food security of the beneficiaries and weak supervision on family’s food selection when using E-cards, and weakness in empowerment and education of mothers were identified as factors that affected the program’s effectiveness [45]. The program implementation costs were estimated at around USD 30 per child (in 2015), mainly related to the food basket and personnel salaries. Implementing the intervention through the primary health care (PHC) system saved the implementation cost [60]. Changes in the policies related to the inclusion of children in the program made it impossible to randomly selecting children in the comparison group in this study [30].

In Pakistan [40], the more significant amount of cash (DC) transferred resulted in a significant reduction in the odds of wasting compared to the SC and FFV groups after six months. However, interventions did not have any effect on wasting at one year. Inability to masking of the intervention to both participants and data collectors because of the process of the evaluation was one limitation mentioned in this study. Conversely, the Conditional Cash Transfer (CCT) in Mexico with a longer duration of exposure (10 years) resulted in improvement of HAZ [61], and double the amounts of cash transferred from approximately USD 800 to USD 1600 was associated with positive effects on stunting reduction and other expected outcomes (e.g., lower prevalence of overweight, motor development, cognitive development, and receptive language) [62]. 

Identification and selection of proper food items where cash or voucher is the distribution mode are important and can show larger effects at the lowest cost if done properly. In this regard, WHO recommends using locally available nutrient-dense foods to prevent children from becoming severely malnourished [63]. The other issue that needs consideration for sustained effect over time is the inclusion of empowerment strategies and eliminating household poverty. A systematic review of the impact of nutrition and cash-based interventions and policies aimed at reducing stunting in LMIC showed that CCT significantly reduces stunting [64]. The conditional nature of the benefits separates CCT programs from other cash or in-kind distribution programs [62]. CCT programs use cash transfers as incentives for parents to invest in their children’s health and wellbeing so that their children will have the capabilities to escape poverty when they reach adulthood [65]. For instance, most CCT programs in Latin America distribute benefits conditional on mandatory attendance at preventive healthcare services and health and nutrition education sessions designed to promote positive behavioral changes. Some programs also require school attendance for school-age children. 

In the present study, a meta-analysis performed on the result of community-based nutrition-specific interventions on child nutritional status demonstrated that the interventions had had a significant effect on HAZ and WHZ of children under 5 compared to the control groups. Sub-group analysis showed that duration of intervention (more than 6 months) was one of factor that modify the effect on WHZ and HAZ, while such an effect was not observed on WAZ. It was shown that nutrition education and cash-based interventions might benefit from more extended implementation periods to affect stunting [64]. 

Besides, the scarcity of available studies in the region and their heterogeneity, make it difficult to conclude one particular type of intervention as the most effective. A combination of nutrition-specific and nutrition-sensitive approaches showed the most significant effect on stunting reduction. Still, there was not necessarily a fixed combination of interventions that demonstrate the most significant impact in all contexts [54]. However, in cases where one particular risk factor is responsible for stunting in most of the population, a simple intervention may have a significant effect. For example, in the regions with a high burden of malaria, prevention and treatment of malaria have been effective in reducing the stunting rate [54]. 

Many systematic reviews have been performed to summarize the much primary research on community-based intervention in U5C in LMIC. However, to our knowledge, this study is the first to consider this issue in the EMR with its specific geographic, political, and economic situations. Despite implementing several programs at the national and sub-national level to improve nutritional status of children in the EMR countries, there is not enough published data regarding their effectiveness and/or cost-effectiveness. For example, baby-friendly hospital initiative (BFHI) has been implemented in 22.7% of the EMR countries [24]. Still there are no qualified RCTs regarding the effect of this program and exclusive breastfeeding on a child’s growth in the region. In addition, considering the high prevalence of child malnutrition in several countries in the region [66,67,68], the limited number of high-quality interventions reported to prevent and/or manage child malnutrition and the limited number of EMR countries focusing on such interventions is alarming. The need for capacity building and promotion of research and policy on finding solutions for child undernutrition should be noted and emphasized. 

Cost-effectiveness is one of the essential factors to consider in program planning and should be measured in pilot interventions, and in the evaluation of ongoing programs. Only one of the included studies had evaluated the cost-effectiveness of different cash-based interventions (in Pakistan) and one had assessed the implementation cost (in Iran). Contacting a responsible person for nutrition in some countries in the region also did not find any data regarding the cost/cost-effectiveness of such interventions. Therefore, more emphasis and proper support for regular and scientifically planned program evaluations addressing these issues are recommended. The other aspects should be considered is to assess intervention outcomes and consider how the implementation process of influences observed outcomes. Causal inferences can be undermined from limitations in the design, data collection, and analysis of primary studies, leading to an underestimation or over estimation of the true intervention effect. Proper strategies, tools, and methods can be used to address the program’s process and implementation. Complementary tools can be applied to make informed judgments about implementation failure [69]. It is highly recommended to pay attention to implementation and process evaluation of the nutrition-specific interventions in the region to provide decision-makers with insights into the conditions needed to generate positive outcomes in the target population. 

Based on the WHO report on global nutrition policy [70], 63% of member states in the WHO-EMR have goals, targets, or indicators regarding wasting, stunting, and exclusive breastfeeding within their national policies. Despite the implementation of different nutrition programs, there are few reports published regarding their effectiveness or cost-effectiveness. In addition, while there are relatively adequate data and published papers available on child nutritional status and some characteristics of the programs implemented in the region, very few high-quality RCTs and effectiveness/cost-effectiveness data is available. Throughout this decade, the regional strategies on nutrition and plan of action have been in place to support Member States strengthen or establish action on nutrition. There have been significant changes in the nutrition landscape. Many countries in the Region have continued to move through the nutrition and epidemiological transition, while other countries have seen increases in undernutrition associated with conflict and political instability. Over the same period, there has also been a series of landmark global and regional commitments to tackle malnutrition in all its forms. To accelerate progress towards the global targets, the United Nations declared a Decade of Action on Nutrition between 2016 and 2025, focusing on six key action areas [71]. 

Variations in the study designs, kind and duration of the interventions, baseline context of the intervention group, targeting method, and differences in the competence and experience of those who delivered the intervention may have resulted in the heterogeneity observed in this review. Besides, there are numbers of limitations in this study that should be taken into account. One limitation is excluding non-RCTs, which may have resulted in the elimination of some other type of studies. Although we tried to gather data from the countries in the region through direct contact, only four countries responded by sending documents on the nutritional status of U5 children based on their national health and nutrition surveys which could not be included in this review. Thus, we have included studies published in peer-reviewed journals and may have missed important data due to publication bias. Additionally, in spite of the fact that Arabic is the official language in most countries in the region, the exclusion of articles published in languages other than English and Farsi may have resulted in language bias. 

## 5. Conclusions

This systematic review showed that in addition to nutrition education and cash-based transfer interventions, there is a need to invest in proper strategies to empower mothers and communities to be more actively involved in such interventions. It was also shown that using PHC capacity will reduce the implementation cost of such interventions and most often facilitates multi-sectoral collaboration and coordination. Considering the high prevalence of child malnutrition in a number of countries in the region, capacity building and investigation regarding the implementation of new approaches and their effect(s) and cost-effectiveness assessment on improving the nutritional status of children are highly recommended. There is a growing body of evidence on the effectiveness, cost‒effectiveness and feasibility of policy interventions to improve nutrition. There is an urgent need to translate this knowledge into action and disseminate lessons from implementation on the ground. Moreover, as guided by the Region Strategy in Nutrition for EMR (2020–2030) [72], there is a need for comprehensive, multi-sectoral action to address malnutrition in all its forms across the Region.

The finding of this study indicates the need for more investment on research in nutrition to enable Member States to report on the Global Targets on Nutrition and SDGs especially SDG2 and SDG3 [3].

## Figures and Tables

**Table 1:** List of excluded articles from the systematic review and the reasons.

Author/Country/Year	Reason for Exclusion
Abolfotouh et al./Egypt/1999 [31]	It was a cross-sectional study and not a community-based intervention
Djazayery A./Iran/2004 [11]	It was a report on the nutrition intervention in the region, there was no data regarding the children’ anthropometry and it was not a randomized trial.
Agha SY./Iraq/2004 [32]	It was a before-after study with no control group.
Jabbari-Beirami et al./Iran/2007 [33]	It was a cross-sectional study and not a community-based intervention
Zavoshy et al./Iran/2012 [34]	It was a before-after study without a control group.
Morikawa et al./Afghanistan/2013 [35]	It was a before-after study with no control group and the primary outcomes were not reported properly.
Lin and Salehi/Afghanistan/2013 [36]	It was an abstract without full text and there were no data regarding children’ anthropometry.
Doocy et al./South Sudan/2013 [37]	There were not any data regarding children anthropometric outcomes.
Cambell L./Lebanon/2014 [38]	Not relevant, as there was not any anthropometric outcome of children, thus the study was beyond the scope of this review.
Soly-maker T/South Sudan/2004 [39]	There was no data regarding children’ anthropometry
Fenn et al., Pakistan/2015 [40]	It was a protocol study
Rouhani et al., Iran/2015 [41]	It was a poster and the full-text was not available.
AL-Mudhwahi A., Yemen/2015 [42]	It was a descriptive study, not a controlled trial.
Ghodsi et al., Iran/2016 [43]	There was no data regarding the children’ anthropometry and it was not a randomized trial.
Zavareh et al., Iran/2017 [44]	A descriptive-analytical study
Ghodsi et al., Iran/2018 [45]	There were not any data regarding children anthropometric outcomes.

**Table 2:** Risk of bias assessment of cluster RCTs.

Author	Selection Bias: Allocation Concealment	Performance Bias: Blinding of Participants and Personnel	Detection Bias: Blinding of Outcome Assessment	Attrition Bias: Incomplete Outcome Data	Reporting Bias: Selective Reporting	Overall RoB Judgment
Ghodsi et al., 2018 [30]	L	S	L	L	L	S
Fenn et al., 2017 [46]	L	S	L	L	L	S
Trenouth et al., 2018 [47]	L	S	L	L	L	S
Forozani, et al., 1999 [48]	L	S	S	S	H	H
Saleem et al., 2014 [29]	L	S	S	L	L	S
Bakhtiari et al., 2017 [49]	H	S	S	H	L	H
Zaman et al., 2008 [50]	L	S	S	L	L	L
Yousafzai et al., 2014 [28]	L	S	L	L	L	L
Yousafzai et al., 2016 [27]	L	S	L	L	L	L
Brown et al., 2017 [51]	L	S	L	L	L	L
Malekafzali et al., 2000 [52]	S	H	H	H	H	H
Sheikholeslam et al., 2004 [53]	H	S	H	H	L	H

RCT: Randomized Controlled Trial; RoB: Risk of Bias, H: High RoB, S: Some concern in RoB, L: Low RoB.

**Table 3:** Characteristics of the included studies reporting the effectiveness of community nutrition-specific intervention(s) for improvement of nutritional status of under five-year-old children in EMR countries: the interventions are categorized into two groups based on their main Methods/strategies.

Author/Year	Country/Region	Design	Type(s) of Intervention	Subjects (n)/ Age/Duration	Outcome(s)
1. Interventions used nutrition education/nutrition counselling					
Zaman et al., 2008	Pakistan/urban	Cluster randomized controlled trial	Mother’s nutrition counselling using locally adapted of Pakistan’s IMCI ‘feeding counselling card’ by Lady Health Workers regarding the recommended foods and frequency of feeding according to age of the child	Mother and 6–24 mo child pairs. Interventions: n = 189 Controls: n = 186/ 6.5m intervention	Baseline: WAZ: I:−1.089 ± 1.22 C: −1.439 ± 1.22, *p* = 0.125 HAZ: I: −1.115 ± 1.36 C: −1.407 ± 1.22, *p* = 0.167 WHZ: I: −0.450 ± 1.01 C: −0.559 ± 1.08, *p* = 0.451 End of the study: WAZ: I: −1.174 ± 1.94 C: −1.720 ± 1.27, *p* = 0.012 HAZ: I: −1.582 ± 1.58 (*p* = 0.55) C: −1.705 ± 1.24 WHZ: I: −0.286 ± 1.22 C: −0.794 ± 1.15 (*p* = 0.004)
Saleem et al., 2014	Pakistan/peri-urban	cluster-randomized interventional trial	Intervention group received maternal educational on importance of breastfeeding (BF), its continuation for the first two years of life and the importance of complementary feeding (CF) initiation at 6 m., age-related complementary food, and hand washing and general hygiene promoting protein-based and iron-rich foods. The control group received advice about BF according to national guidelines	Mothers of infants aged 10–20 weeks. Interventions: n = 118 Controls: n = 94/ 6 m intervention	Intervention group had a higher mean weight of 350 g (*p* = 0.001); and length of 0.66 cm (*p* = 0.001) (*p* = 0.002) compared to the controls; proportionate reduction of stunting and underweight were 10% (84% vs. 74%; OR adj 8.36 (5.6–12.42) and 5% (25% vs. 20%; OR adj 0.75 (0.4–1.79) in the intervention compared to the control group.
Yousafzai et al., 2014 (Yousafzai et al., 2014)	Pakistan/rural	factorial, cluster-randomized effectiveness trial	Pakistan Early Child Development Scale-Up (PEDS): 1: Enhanced Nutrition (EN) group received nutrition education regarding association of good nutrition and health, additional advice on responsive feeding, and problem solving about feeding and a multiple micronutrient powder which contains iron, folic acid, Vit A, and Vit C. 2: Responsive Stimulation (RS) group: a local adaptation of the Care for Child Development approach developed by UNICEF and WHO) 3.Combined responsive stimulation and enhanced nutrition (RS&EN) 4. Controls: routine health and nutrition services	Infants up to 2.5 month divided to four groups and received intervention till age 24 m: EN: n = 364 RS: n = 383 RS&EN: n = 374 Controls: n = 368/Up to 24 m.	HAZ 6 m: EN: −1.2 ± 1.3 vs. no EN: −1.4 ± 1.2, *p* < 0.0001; 18 m: EN: −2.2 ± 1.2 vs. no EN: −2.4 ± 1.1, *p* = 0.02. Not significant differ in mean WAZ between the groups at 6, 12, 18, or 24 m.
Yousafzai et al., 2016	Pakistan/rural	Follow-up study of the initial cluster-randomized effectiveness trial	Pakistan Early Child Development Scale-Up (PEDS): 1: Enhanced Nutrition (EN) 2: Responsive Stimulation (RS) 3: Combined responsive stimulation and enhanced nutrition (RS&EN) 4: Controls (C)	1302 mother–child dyads (87% of the dyads in the original enrolment) followed up at 4 years of age 1-EN: n = 311 2-RS: n = 345 3-RS&EN: n = 315 4-C: n = 331 testing differences between exposures to the two interventions (RS (n = 657) vs. no RS (n = 638) and EN (n = 620) vs. no EN (n = 675))/ 4 years follow up study	RS (data are mean (%95CI)): WAZ: 0.8 (−0.9 to −0.7) HAZ: −0.9 (−1.0 to −0.7). WHZ: −0.5 (−0.5 to −0.4) no RS WAZ: −0.9(−0.9 to −0.8) HAZ: −0.09(−1.0 to −0.7) WHZ: −0.5 (−0.6 to −0.4) EN: WAZ:−0.8 (−0.9 to −0.7) underweight: 11% HAZ: −0.8 (−1.0 to −0.7) WHA: −0.4 (−0.5 to −0.3) No EZ: WAZ: −0.9 (−1.0 to −0.8) HAZ: −0.9 (−1.0 to −0.8) WHZ: −0.5 (−0.6 to −0.4)
Brown et al., 2017	Pakistan/rural	factorial, cluster-randomized effectiveness trial	Pakistan Early Child Development Scale-Up (PEDS): 1: Enhanced Nutrition (EN) 2: Responsive Stimulation (RS) 3: Combined responsive stimulation and enhanced nutrition (RS&EN) 4: Controls (C)	1302 mother–child dyads at 4 years of age 1-EN: n = 364 2-RS: n = 383 3-RS&EN:n = 374 4-C: n = 368/ 2 years (indicators related to measures at ages 2 and 4)	Mother–child interaction quality mediated the effects of both EN and RS on HAZ at 4 years via its longitudinal stability from 2 years of age (β = 0.016, *p* = 0.005; β = 0.048, *p* < 0.001, respectively).
**2. Interventions used food distribution through different modalities**					
Fenn et al., 2017	Pakistan/rural area	Cost, cost-efficiency, and cost-effectiveness study, cluster randomized controlled trial	Research on Food Assistance for Nutritional Impact (REFANI): Monthly cash-based interventions (CBIs) for 6 months: 1: Standard cash (SC) (PKR 1500, USD 14) 2: Double cash (DC), (PKR 3000); 3: Fresh food voucher (FFV) of PKR 1500 (USD 14, could be exchanged for specified fresh foods (fruits, vegetables, milk, and meat) in nominated shops)	6–48mo.children Controls: n = 852 DC: n = 839 FFV: n = 866, SC: n = 905/ 6 mo. CBIs distribution and follow-up on 12 mo	Mean WHZ significantly improved in both the FFV and DC arms at 6mo (FFV: z-score = 0.16; 95% CI 0.05, 0.26; *p* = 0.004; DC: z-score = 0.11; 95% CI 0.00, 0.21; *p* = 0.05) compared to the CG. All three intervention groups showed similar significantly lower odds of being stunted (HAZ < −2) at 6 mo (DC: OR = 0.39; 95% CI 0.24, 0.64; *p* < 0.001; FFV: OR = 0.41; 95% CI 0.25, 0.67; *p* < 0.001; SC: OR = 0.36; 95% CI 0.22, 0.59; *p* < 0.001) and at 1 y (DC: OR = 0.53; 95% CI 0.35, 0.82; *p* = 0.004; FFV: OR = 0.48; 95% CI 0.31, 0.73; *p* = 0.001; SC: OR = 0.54; 95% CI 0.36, 0.81; *p* = 0.003) compared to the CG.
Ghodsi et al., 2018	Iran/urban and rural area	Quasi-experimental and mixed-methods	Monthly in-kind food/food voucher/cash transfer for buying specified food items (cheese, honey, spaghetti, oil, sugar, bean, lentil, eggs, milk, and chicken) from nominated shops	under 5yr. child Controls: n = 409, Interventions: n = 362/ 6 m intervention	Significant differences (*p* < 0.001) were seen within each group in each index at the end of the study. Mean difference of indices in Intervention group after 6 mo.: WAZ: 0.16 ± 0.62 HAZ: 0.08 ± 0.77 WHZ: 0.2 ± 0.91 Mean of difference in controls: WAZ: 0.13 ± 0.68 HAZ: 0.07 ± 1.04 WHZ: 0.2 ± 0.81
Trenouth et al., 2018	Pakistan/rural area	Cluster randomized controlled trial and mixed-methods process evaluation	Research on Food Assistance for Nutritional Impact (REFANI): Monthly cash-based interventions (CBIs): 1: Standard cash (SC) (PKR 1500, USD14) 2: Double cash (DC),(PKR 3000; 3: Fresh food voucher (FFV) of PKR 1500 (14 USD, could be exchanged for specified fresh foods (fruits, vegetables, milk, and meat) in nominated shops)	6–48 mo. children Controls: n = 852 DC: n = 839 FFV: n = 866, SC: n = 905/ 6 mo. CBIs distribution and follow-up on 12 mo.	TCTRs: DC: USD 1.82, SC: USD 2.82, and FFV: USD 2.73. The total cost per beneficiary household: SC: USD 193, FFV: USD 220, and DC: USD 284 Operational costs per recipient household: SC: USD 105, DC: USD 109, and FFV: USD 132 The cost per child in a household receiving cash or vouchers: DC: USD 203, SC:USD 135, FFV: USD 160 The cost per case of stunting averted: DC: USD 1290, SC: USD 882, FFV: USD 883. The cost per DALY averted *: DC: USD 64, SC: USD 434, and FFV: USD 563

EMR: Eastern Mediterranean Region; CG, control group; CI, Confidence interval; DC, double cash; FFV, fresh food voucher; OR, odds ratio; SC, standard cash; CBIs: cash-based interventions; DALY: Disability adjusted life year; EN: Enhanced Nutrition; RS: Responsive Stimulation WHZ, weight-for-height *z*-score; WHZ, weight-for-age *z*-score, WAZ; Height—for-Age, HAZ. OR_adj_: Adjusted for age in days at baseline, vaccination status at baseline, father’s and mother’s education, TCTR: Total cost transfer ration; *: without discounting or age weighting.

## Data Availability

No new data were created or analyzed in this study. Data sharing is not applicable to this article.

## Supplementary Materials

The following are available online at https://www.mdpi.com/article/10.3390/ijerph18157844/s1. Supplementary File S1. PRISMA Checklist.
